# Recent Advances and Challenges in Textile Electrodes for Wearable Biopotential Signal Monitoring: A Comprehensive Review

**DOI:** 10.3390/bios13070679

**Published:** 2023-06-26

**Authors:** C. M. Vidhya, Yogita Maithani, Jitendra P. Singh

**Affiliations:** Department of Physics, Indian Institute of Technology Delhi, Hauz Khas, New Delhi 110016, India

**Keywords:** wearable electronics, textile electrode, dry electrodes, smart textiles, electrocardiogram, electromyogram, biopotentials

## Abstract

The technology of wearable medical equipment has advanced to the point where it is now possible to monitor the electrocardiogram and electromyogram comfortably at home. The transition from wet Ag/AgCl electrodes to various types of gel-free dry electrodes has made it possible to continuously and accurately monitor the biopotential signals. Fabrics or textiles, which were once meant to protect the human body, have undergone significant development and are now employed as intelligent textile materials for healthcare monitoring. The conductive textile electrodes provide the benefit of being breathable and comfortable. In recent years, there has been a significant advancement in the fabrication of wearable conductive textile electrodes for monitoring biopotential signals. This review paper provides a comprehensive overview of the advances in wearable conductive textile electrodes for biopotential signal monitoring. The paper covers various aspects of the technology, including the electrode design, various manufacturing techniques utilised to fabricate wearable smart fabrics, and performance characteristics. The advantages and limitations of various types of textile electrodes are discussed, and key challenges and future research directions are identified. This will allow them to be used to their fullest potential for signal gathering during physical activities such as running, swimming, and other exercises while being linked into wireless portable health monitoring systems.

## 1. Introduction

Wearable biopotential signal monitoring is a promising technology that has gained significant attention in recent years owing to its potential to revolutionize healthcare by enabling continuous and non-invasive monitoring of human physiological signals. Instead of the cumbersome healthcare systems used in hospitals, it is now possible to monitor patients’ health at home at their convenience and to improve healthcare facilities and treatment in separate locations due to the miniaturization of electronic devices [1,2]. Wearable electronics make it feasible to track patients’ health status and keep an eye on their medical issues over a prolonged length of time. Wearables can be used to treat neuromuscular ailments, fatal cardiovascular conditions, muscle-building for sports, and anomalies connected with the brain and at rehabilitation facilities [3,4,5]. Cardiovascular disease, the major cause of death across the globe, in which the ischemic disease, which is the leading cause of cardiovascular disease, alone was responsible for 9.44 million deaths in 2021 [6]. The early detection and management of CVD can significantly improve patient health and reduce the medical expenses. Hence, wearable biopotential signal monitoring are an an effective tool for early detection and monitoring of CVD as well as other health conditions, such as sleep disorders, epilepsy, and Parkinson’s disease [7,8,9].

Previously used only for clothing, textiles have transformed into smart textiles, which have a variety of applications, including temperature, pressure, strain, and motion sensing [10,11,12,13]. Due to the comfort that clothing offers, it may be simpler if the measuring device is incorporated into the clothing so that patients can wear it without any restrictions and without the need for special skin preparations [14]. Additionally, textile materials are freely accessible, which lowers manufacturing costs. Due to their distinctive qualities, including flexibility, breathability, and washability, textile electrodes are a promising technology for wearable biopotential signal monitoring [15,16]. Reusable electrodes will be more economically feasible for the long-term monitoring of biopotentials. Textile electrodes are therefore preferable to commercial wet electrodes made of Ag/AgCl that cannot be reused. Since the gel in commercial wet electrodes might get dehydrated and reduce the signal quality, they cannot be utilised for extended periods of time [17].

The development of textile electrodes still faces challenges, such as the need to enhance their signal quality and durability and deal with problems with the skin–electrode contact. One of the main challenges is the requirement to enhance the signal quality of textile electrodes, which are impacted by a number of parameters, including electrode–skin interface impedance, motion artefacts, and noise interference [18,19]. Enhancing the durability of textile electrodes, which experience repetitive mechanical stress during usage and washing, is another problem [20]. Recent studies have concentrated on these issues, and great progress has been achieved in the creation of textile electrodes for biopotential signal monitoring.

Wireless healthcare technology has emerged as a key enabler for remote patient monitoring and telehealth services, providing opportunities for continuous and personalized healthcare services outside the traditional clinical settings. With the increasing prevalence of chronic diseases and aging populations, wireless healthcare technology has become increasingly important for enhancing the quality of care [21,22]. As such, this review paper focuses on the recent advances and challenges in textile electrodes for wearable biopotential signal monitoring as one of the key wireless healthcare technologies, highlighting their potential for improving healthcare outcomes and addressing the challenges faced in this field.

This comprehensive review paper provides an in-depth systematic overview of biopotentials with the main focus on electrocardiography (ECG) and electromyography (EMG). It reviews the evolution and recent advances in textile electrodes for wearable biopotential signal monitoring, including materials and fabrication techniques, signal quality enhancement methods, and integration with wearable devices. Furthermore, the review paper highlights the current challenges and future directions in this field, providing insights for researchers, clinicians, and industry professionals working in this area.

## 2. Biopotentials

Biopotentials are electrical signals produced by excitablecells like the neurons and cardiac cells and tissues as a result of the movement of charged particles like ions across cell membranes. The difference in electrical potential between the interior and the exterior of the cell as a result of ion transport across the cell membrane generates the electrical signals which are measurable in millivolts. These electrical potentials known as biopotentials travel towards the body’s surface, which serves as a conductor of volume [23,24]. Using electrodes positioned on the skin or directly on the tissue of interest, this electrical potential can be recorded. In disciplines such as cardiology, neurology, and sports science, biopotentials are frequently employed for diagnostic and research purposes since they can reveal important information about the physiological status of cells and tissues [25]. Collecting and analysing these signals help in understanding of the functioning of the body’s organs and abnormalities associated with them.

**Figure 1:**
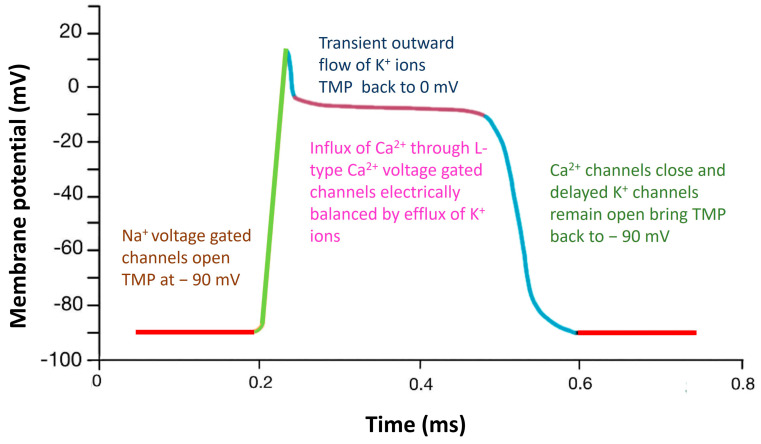
Action potential curve which illustrates the change in membrane potential during ion diffusion in cardiac myocytes.

The movement of sodium, potassium, and calcium ions across the voltage-gated channels are responsible for the propagation of action potentials through the heart and muscles. The resting membrane potential of −90 mV is raised to more positive values during depolarization, and the cell potential value is restored during repolarization [26]. The mechanism of voltage mediated ion transport across the cell membrane, resulting in the action potential curve in the depolarization–repolarization cycle, is illustrated in Figure 1. 

### 2.1. Electrocardiogram (ECG)

**Figure 2:**
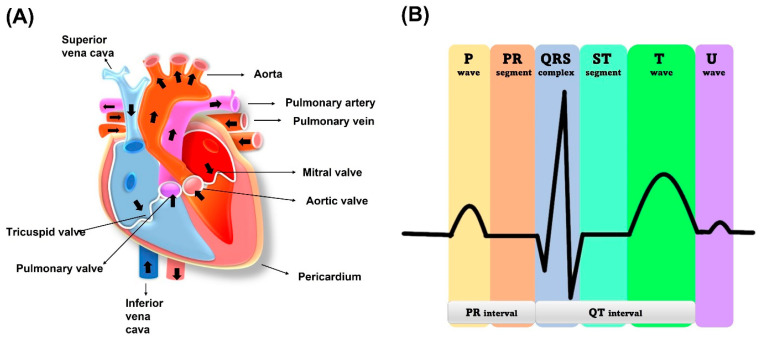
(**A**) Human heart schematic representation. (**B**) ECG standard waveform from human heart.

The human heart is a vital organ that pumps blood to the entire body. It is divided into four chambers, with the two upper chambers called atria and the two lower chambers called ventricles, as shown in Figure 2. Blood flows from the atria to the ventricles through an atrioventricular septum, which separates them. The heart’s contraction is what moves blood throughout the body. The muscles surrounding the atria and ventricles contract, squeezing the blood down and out of the heart. The heart has valves that ensure blood flows in one direction. The tricuspid valve is between the right atrium and right ventricle while the mitral valve regulates blood flow between the left atrium and left ventricle. Blood is received by the heart through two main veins, the superior vena cava and inferior vena cava, which bring in deoxygenated blood from the body. After the blood is pumped out of the right ventricle, it travels to the lungs through the pulmonary valve and artery. At the lungs, blood is oxygenated and returns to the left atrium via the pulmonary vein. The left ventricle then pumps the oxygenated blood throughout the body. The cardiac muscle in the left ventricle wall is thicker than the right ventricle wall because it needs to pump blood throughout the entire body [27].

The contraction of the heart is a crucial step in the circulation of blood throughout the body. This contraction process is controlled by the propagation of electrical signals induced by cation exchange through the heart cells [28].

The electrical activity of the heart that generates the ECG signal is produced by the depolarization and repolarization of cardiac muscle cells. When the heart muscle cells depolarize, they become more positively charged, creating a small electrical current that can be detected on the surface of the skin. The sinoatrial (SA) node is known as a natural pacemaker of the heart. The SA node is located in the wall of the right atrium, as shown in Figure 2. This electrical activity produces the P wave, generation of electrical potentials by atrial contraction, QRS complex, electrical potentials by ventricular contraction, and T wave due to the ventricles returning to their normal state after contraction. To measure the ECG signal, electrodes are placed on the skin over the heart. These electrodes detect the electrical activity of the heart and send it to an amplifier, which amplifies the signal and sends it to a recording device. The recorded signal can then be analysed to diagnose various heart conditions [29]. The ECG signal is a result of the difference in electric potential between two points on the body. The potential difference is caused by the movement of charged particles, specifically ions, across the cell membrane of the heart muscle cells. This movement of ions is due to the opening and closing of ion channels in the cell membrane in response to various stimuli.

The electrical activity of the heart can be described in terms of the principles of electrophysiology. This field of study examines the electrical properties of biological cells and tissues and how they relate to the function of the body. The principles of electrophysiology are based on the laws of physics, including Ohm’s law, which describes the relationship between current, voltage, and resistance, and the principles of electrical capacitance and inductance. By applying these principles, researchers can gain a better understanding of the electrical activity of the heart and how it can be measured and analysed to diagnose heart conditions [26].

The amplitude of ECG signals ranges from 1–5 mV [24]. If there are any abnormalities associated with the functioning of the heart, it can be observed from the altered ECG pattern. For example, when there is an AV node block, there is a delayed transmission of the electrical impulse from atria to ventricles leading to a longer PR interval in the ECG pattern, and the absence or broadening of certain patterns also indicates the abnormal functioning of the heart [26].

### 2.2. Electromyogram (EMG)

**Figure 3:**
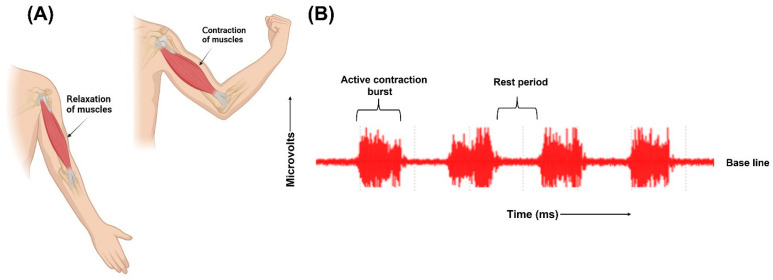
(**A**) Representation of contraction and relaxation of muscles. (**B**) Graphical representation of raw EMG signals.

The human body has both smooth and skeletal muscles. The skeletal muscles are responsible for the movements of the body. The skeletal muscles are composed of numerous fibres. Each of these fibres are made of smaller subunits called myofibrils ranging from hundreds to thousands. Each myofibril is composed of many myosin filaments, which are large, polymerized protein molecules responsible for the actual muscle contraction. These nerve fibres innervate the skeletal muscle fibres, and the action potential is initiated in the muscle fibre by the nerves. A single motor nerve fibre and all the muscle fibres it innervates comprise a motor unit. The origin and execution of muscle contraction begins when the action potential travels along a motor nerve to its ending on the muscle fibres. Neurotransmitter acetylcholine is released at the nerve ending. This leads to the diffusion of large quantities of sodium ions into the interior of the muscle fibre membrane through the acetylcholine-gated channels. Depolarization occurs, leading to opening of voltage-gated sodium channels initiating action potential at the membrane. It depolarizes the muscle membrane, and action potential electricity flows through the centre of muscle fibre. Calcium ions are released from the sarcoplasmic reticulum, which initiates attractive forces between the actin and myosin filaments, causing them to contract. After a fraction of a second, the calcium ions are pumped back and stored until a new muscle action potential is initiated. The removal of calcium ions causes the relaxation of the contracted muscles. This depolarization–repolarization cycle forms an electric dipole and travels through the surface of the muscle fibre. The movement of ions generates electrical potential in the muscle cells which travels along the surface of muscle fibre [26]. Electrodes placed on the surface of the skin records this electrical activity. The electrodes detect the superposition of all active motor units that can be detected under the bipolar electrode configuration site. Recording the electrical activity of the muscles and using it for clinical examination or for athletics training is called electromyography [25,30]. Figure 3 illustrates the contraction and relaxation of muscles and the recording of raw EMG signals.

The signals recorded by the electrodes are raw, unfiltered EMG signals. Relaxed muscles are recorded as a less noise-free EMG baseline while on the contraction of the muscles, the signals are recorded as random non-reproducible amplitude spikes since motor units detected each time by the electrodes are not constant. Raw EMG signals range from −5 millivolts to 5 millivolts with a frequency range between 6 Hz to 500 Hz [30].

Recording EMG signals can help in understanding muscle fatigue, which is the decrease in the maximum contraction capability of specific muscles [31]. For a subject with muscle fatigue, there is a decrease in the fibre conduction velocity, with a diminishing number of fast contracting motor units. This results in shift of EMG power spectrum towards lower frequencies and the amplitude of the signals can increase because of the recruitment of additional motor units and their growing firing rates to sustain the required force. Textile electrodes are successful in monitoring EMG muscle fatigue monitoring. Graphene textile electrodes, which were fabricated with a dip–dye–reduce cycle on nylon fabric, were sewn onto elastic bands, which ensure sufficient pressure for skin electrode coupling. The textile electrodes placed on bicep brachii muscles successfully recorded the signals shifted to a lower frequency and increased amplitude, proving textile electrodes have the potential to be used in muscle fatigue monitoring, replacing the wet electrodes [32]. To confirm that textile electrodes have the potential to be used instead of commercial wet electrodes, the performance of the textile electrode was analysed in terms of the signal-to-noise ratio, variation in signal amplitude, medium frequency, and signal quality of dynamic movement. The textile electrodes were placed at the deltoid muscle. The EMG signals obtained from both textile and dry electrodes were comparable, with no significant difference in the median frequency and SNR ratio observed [33].

Biopotentials such as ECG monitoring can help detect abnormalities in the heart’s electrical activity, which can indicate conditions such as arrhythmia or myocardial infarction [34,35]. This information can be critical in making diagnoses and determining appropriate treatment options for patients. EMG monitoring can help detect abnormalities in the electrical activity of skeletal muscles, which can indicate conditions such as muscular dystrophy or nerve damage [36,37]. This information can also be used to guide treatment options and track the progression of these conditions. Overall, biopotential monitoring is an important tool in understanding the function of the human body and detecting abnormalities that can impact a person’s health and wellbeing to improve their health and to reduce death rates by providing timely treatments.

## 3. Biopotential Electrodes

Biopotential electrodes are used to record the biopotential signals produced by different organs. Since the flow of electrical current inside the body is due to the ions, they need to be converted into electrons to be measured by the electrical circuit. Biopotential electrodes act as transducers, converting the ions from body to electrons at the skin electrode interface by electrochemical reactions, allowing the flow of the current from the human body to the monitoring device [38].

### 3.1. Different Categories of Biopotential Electrodes

**Figure 4:**
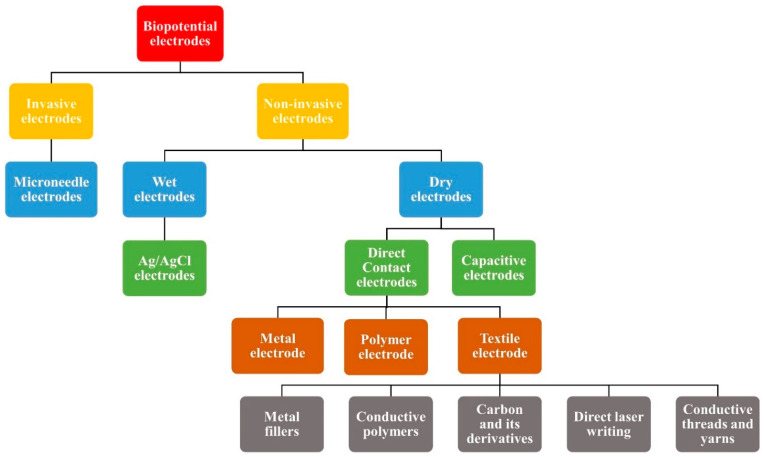
Classification of different types of biopotential electrodes.

There are several types of biopotential electrodes depending upon the skin to electrode interaction, such as invasive and non-invasive electrodes. Invasive electrodes such as needle electrodes are typically used for deeper biopotential measurements such as intramuscular EMG. They penetrate the stratum corneum layer of the skin, which is the most resistive layer of the skin and hence the skin contact impedance is very low [39]. Different types of non-invasive electrodes are also used for ECG and EMG signal analysis. Surface electrodes used for non-invasive biopotential monitoring can be categorized into wet and dry electrodes, based on their charge transfer mechanism with the skin. Figure 4 represents the various kinds of biopotential electrodes.

### 3.2. Wet Electrodes

Wet electrodes have a layer of gel between the electrode and skin interface. They are non-polarizable electrodes, where there is a charge transfer occurring in the electrolytic medium from chemical reactions [24,40]. The chemical reaction that occurs at the electrode–electrolyte interface for charges to cross the interface can be represented as follows.

C ⇋ C^n+^ + ne^−^ (1)

A^m−^ ⇋ A + me^−^ (2)

The above reactions represent the oxidation dominating at the electrode–electrolyte interface. The atoms of the metal can get oxidized, and they give up their electrons, which goes to the electrode while the atoms oxidized enter the solution as cations. In addition, the cations coming from the gel can become oxidized and then give their electrons to the electrode while depositing on the electrode as a neutral atom. Reverse reactions also occur at the interface. These oxidation and reduction reactions are responsible for the transduction of an ionic current to electronic current at the electrode skin interface [38].

**Figure 5:**
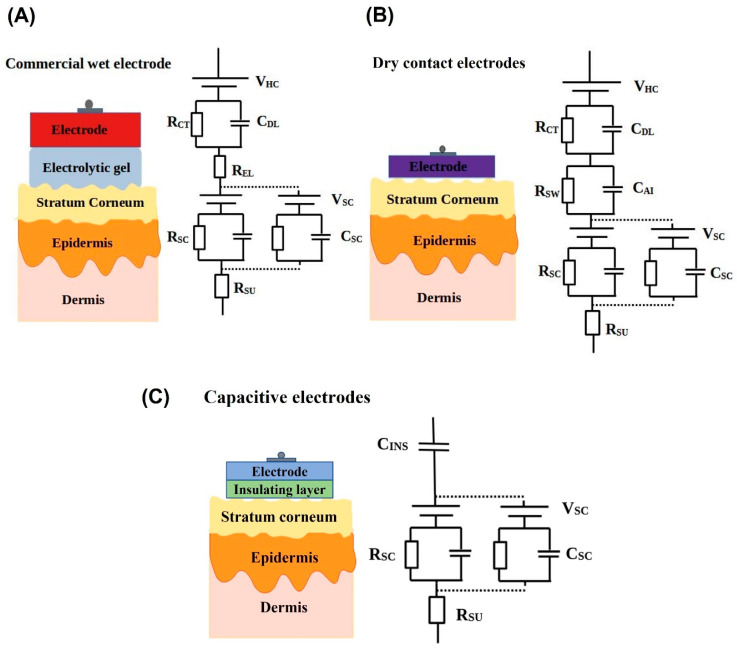
Schematic representation of electrical equivalent models of (**A**) wet electrodes, (**B**) dry contact electrodes, (**C**) capacitive electrodes.

When the electrode comes in contact with the electrolyte there is a spatial distribution of charges forming an electrical double layer. This charge distribution gives a potential at the metal electrolyte interface different from rest of the solution and this potential difference is called the half-cell potential V_HC_ [38]. The interface between the electrode, electrolyte and the skin can be represented by equivalent electrical models. The capacitance of the electrical double layer is represented by C_DL_, and the leakage resistance across this double layer is given by R_CT_. Both C_DL_ and R_CT_ are parallel to each other. The variation in charge in the electrolyte from the metal interface is represented by a series resistance R_EL_. The skin is primarily divided into three layers, which are the topmost layers, the epidermis; and the conductive layers, the dermis; and the subcutaneous layers. The topmost layer of the epidermis is the stratum corneum, which is highly insulating and is sandwiched between the electrolyte and the other conductive layers of the skin. This acts as a dielectric layer, which is modelled by a capacitance C_SC_. The presence of hair follicles, sweat glands, and pores in the stratum corneum adds a leakage resistance R_SC_, and the dermis and subcutaneous layers below the stratum corneum act as a conductive media, having a resistance of R_SU_. The electrolyte–electrolyte interface forms a potential difference called the liquid junction potential represented by V_SC_ [40]. The electrical equivalent model for electrode, electrolyte, and skin interface for the wet electrodes is represented in Figure 5A.

The good signal quality, the lower skin contact impedance, and the low cost makes the wet electrodes the best choice for clinical settings in health monitoring. Commercially used wet electrodes contain silver, which is a highly conductive metal, interfaced to its salt, silver chloride, and a gel rich in ions of silver chloride. These electrodes have the lowest and stable junction potential. Due to the sticky nature of the gel, the electrode stays in position on the skin, which leads to reduced motion artefacts. Hence, the signals will be less distorted [24], but this electrode has various limitations. The electrode has a smaller shelf life [41]. They can be used only for the short-term monitoring of the biopotential signals, such as ECG and EMG. Some patients suffering certain heart conditions may need the long-term monitoring of the ECG; in such cases, when these electrodes are used, the gel present can get dehydrated, which can result in poor signal quality. Moreover, some people can have irritation or itchiness on their skin while using the Ag/AgCl electrode [42]. All these have motivated researchers into looking upon on an alternative electrode that can overcome the shortcomings of the wet electrodes.

### 3.3. Dry Electrodes

In the dry electrodes, there is no layer of gel between the electrode and skin interface. One of the advantages of the dry electrodes is that they can be used for long-term monitoring. This eliminates the problem of gel dehydration and loss is signal quality [43]. In addition, the problem of skin irritation can be avoided. Generally, when using textile-based dry electrodes, they have the advantage of being washable and reusable. Using textile- and polymer-based dry electrodes have the advantage of a better skin conformance due to their flexible nature [44,45], but one of the challenges of using dry electrodes is a higher skin contact impedance due to the absence of a gel layer. However, the presence of sweat accumulated between the electrode skin interface reduces the skin contact impedance [46]. There can be high signal-to-noise (SNR), and they are prone to motion artifacts [40]. Wet electrodes typically require skin preparation. Before placing wet electrodes, it is normal practice to shave and clean up with alcohol. Researchers are extensively examining ways to overcome these challenges, utilising dry electrodes without gel, adhesives, or skin preparation in general. This indicates the benefit of dry electrodes over wet electrodes in getting around the extra stages of skin preparation [47].

#### 3.3.1. Direct Contact Electrodes

Dry contact electrodes have direct contact with the skin surface. The absence of gel leads to a high skin contact impedance. Different kinds of direct contact dry electrodes are metal electrodes, contact penetrating electrodes, polymer electrodes, and textile electrodes [48]. Electrodes with microneedles can overcome the challenge of a high skin contact impedance since their microneedle array at the surface of the electrode can penetrate the stratum corneum, which is highly insulating in nature [49]. Metal electrodes have better conductivity, are durable, and can be reused, but they are rigid and do not have good conformance with the skin. This can lead to the movement of the electrodes when the patient moves and can result in distorted signals and low SNR [46]. Taking into consideration for the importance of the skin conformance of the electrode for a reduced motion artefact and good signal quality, flexible dry electrodes were investigated. Polymer and textile electrodes are good choices of the materials, which can give better skin conformance. Various types of polymer materials, such as PDMS and polyurethane, which has good biocompatibility and Young’s modulus, comparable to skin and flexibility have been used for the fabrication of dry electrodes [50,51]. The advantage textile electrodes give over the polymer electrodes is the breathability due to its porous nature and user comfort. It is easy to integrate the electrode into daily wearable clothing [52,53]

The electrical equivalent model for the electrode skin interface for the dry contact electrode is represented in Figure 5B. In dry electrodes, instead of the gel electrolyte, moisture and sweat act as the electrolyte, which lowers the skin contact impedance, but since it is not uniform and has less ionic concentration than that of gel electrolyte, the contact impedance can be higher than that of wet electrodes and can vary over time. In addition, since there will be a gap between the electrode and skin, this can add a strong capacitive component to the skin contact impedance. This is represented by C_AI_ in the electrical equivalent model of the dry electrode. The half-cell potential is given by V_HC_. The sweat accumulated in the electrode–skin interface are not rich in ions like the gel and are unevenly distributed. At the electrode–sweat interface, due to a double layer capacitance by charge distribution, there is a capacitance and resistance C_DL_ and R_CT_. R_SW_ in the series represents the resistance due to the sweat layer. In the absence of gel, if the electrode is not completely adhesive to the skin, they can trap air bubbles between the electrode and the skin, giving a capacitive behaviour C_DL_ [40].

#### 3.3.2. Capacitive Electrodes

A capacitive electrode is a category of dry electrodes, which have an insulating material between the electrode and the surface of the skin. The conductive electrode and the surface of the skin separated by a dielectric or insulating material give rise to a capacitive component. These electrodes record the biopotential signals by capacitive coupling of the electric displacement current [54]. The electrical equivalent model of the capacitive dry electrodes is given in Figure 5C. The capacitance between the electrode and the skin is modelled as C_INS_. There is an absence of electrode–electrolyte interface. The potential and the impedances offered by the stratum corneum and conductive layers below it is modelled as shown in Figure 5C.

**Figure 6:**
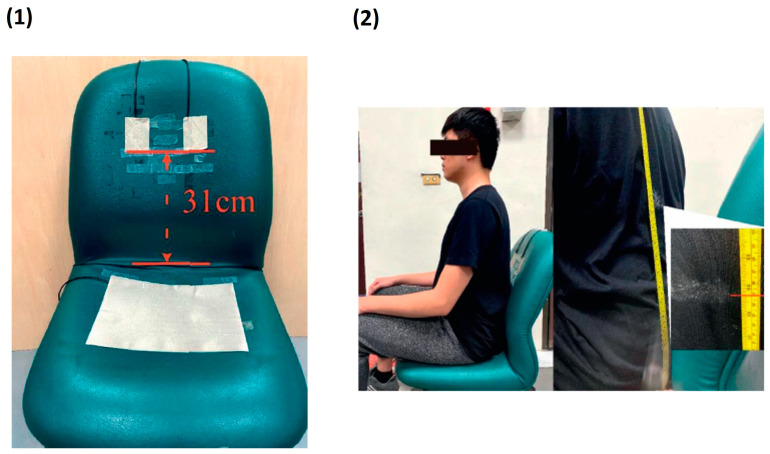
Capacitive electrodes (**1**) conductive textile placed on a chair (**2**) relative position between the participant and the textile electrode placed on the chair for ECG monitoring. Adapted with permission from [58]. Copyright 2021, Su P et al., Published by Hindawi.

The main advantage of using a textile-based capacitive electrode is that there is not direct contact of the electrode with the skin. Therefore, this prevents problems like allergy, skin irritation, etc. that were observed in the contact electrode. In addition, this kind of electrode provides comfort to the user during long-term measurements [55]. Some of the disadvantages associated with capacitive electrodes are high skin contact impedance and high motion artefacts and noise in the signal. In addition, the skin contact impedance can vary according to the movement of the subject [56]. Therefore, a preamplifier can be used, and the electrode (except for the contact surface) can be shielded to reduce the noise [57]. There has been a successful usage of dry capacitive electrodes for biopotential signal measurements. Conductive electrodes can be embedded in objects used day-to-day to comfortably monitor ECG signals. Conductive textile electrodes were placed on a chair to measure the ECG of the patient in a non-contact way, given by Figure 6, where the conductive textile and the human body are considered the two plates of a parallel plate capacitor with cloth in between acting as an insulating layer. Signal processing was performed, and the suitable positioning of the electrodes and the factors affecting the reading was analysed to get better ECG waveforms [58]. ECG monitoring during human sleep can be performed by non-contact conductive textile electrodes. To improve the coupling capacitance caused by the low dielectric constant of bed sheets and pajamas, conductive hydrogel layer P(SBMA-co-HEMA) was applied in an array pattern onto the textile. This lowered the impedance and improved the quality of ECG signals. Eight pressure sensors were integrated to capture sleep postures, and ECG signals were detected from different sleep postures [59].

The advantage of employing textile electrodes for ECG and EMG monitoring is the comfort the textile offers on the skin compared to other dry electrodes like metal or polymer varieties. Making them a part of daily attire makes it simpler to measure the signals. Even while sweat and moisture are needed to lower the skin contact impedance, excessive sweat that gets trapped between the skin and electrode might irritate the user and make the wearer uncomfortable. Additionally, it may cause the electrodes to acquire signals that are unstable and inaccurate [60]. The hydrophilic nature of textiles causes some perspiration to be retained in them. This may assist in lowering the skin contact impedance, a significant problem with dry electrodes. Extra sweat might drain thanks to the fabric’s porosity [61]. The electrode’s biocompatibility is also significantly influenced by the production process and the conductive materials employed. A hydrophilic and aqueous ink was created utilizing graphene and silk sericin to solve the water absorption issues brought on by the hydrophobic nature of graphene. This conductive ink was used to create a washable, breathable, and moisture-permeable textile electrode for the capture of human signals [62]. The conductive textile electrodes are mostly made of silver and other carbon-based materials, such as graphene, reduced graphene oxide, and conductive polymers. These substances induce neither allergic reactions nor irritation in human skin when exposed to it for an extended period of time [56,63]. The use of cloth electrodes makes it feasible to avoid the skin irritants often present in wet electrode adhesives [64]. Conductive electrodes are simple to include in clothing. Embroidering conductive threads and yarns in the appropriate places on the clothing is one quick and easy way to create conductive fabrics [65]. This technique allows for the creation of customized wearable clothing by pinpointing the precise locations for the electrode embroidery. Other techniques include knitting or stitching with conductive threads on finished clothing. Even while hand sewing is a simple procedure, it can compromise the electrode’s stability and durability [66]. The finished clothes can simply have textile electrodes that have already been made using conductive coatings affixed to them. The elastic hand sleeves or leg sleeves can also be used to attach the textile electrodes. They can also be fastened to the wrist using elastic bands and Velcro straps [67,68,69]. In Section 4 and Section 5, which follow, a detailed description of various coating and fabrication techniques to create conductive fabrics is discussed.

## 4. Different Conductive Coatings on Textile

### 4.1. Metallic Coating

**Figure 7:**
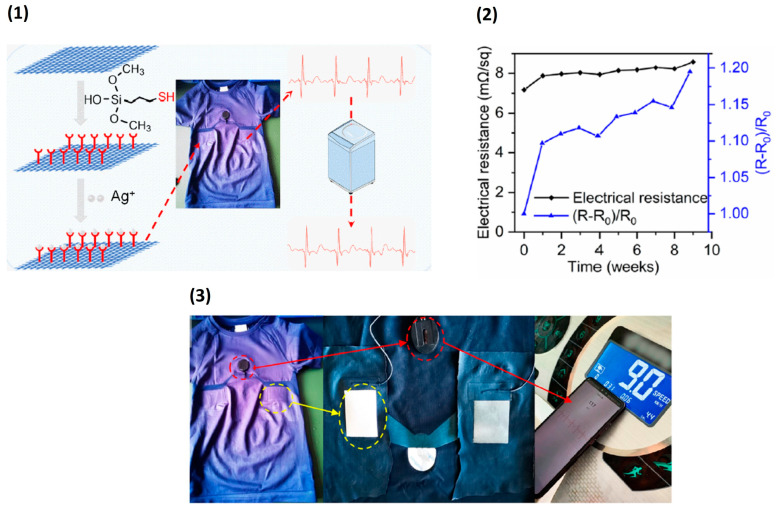
Metal coated electrodes (**1**) an illustration of grafting thiol groups and adding silver by electroplating to fabric for surface modification and the stability of fabricated electrodes in acquiring ECG signals after washing (**2**) changes in the electrical resistance of modified Ag/modified polyester fabric (M-PETF) under tensile condition (**3**) smart garments with the fabricated conductive textile electrodes and the signal capturing and transmitting data to a smart phone. Adapted with permission from [75]. Copyright 2022, American Chemical Society.

Metals, such as copper, silver, and steel, are commonly used in smart textile applications to make conductive textiles due to their high electrical conductivity and biocompatibility. While there are many types of fabrics that can be combined with metal, the durability of metal-coated fabrics can be a concern when they are worn or washed because they may peel off easily from the areas that are exposed to air or water [70,71]. The conductive textile should withstand a certain number of washing cycles; the conductive layer should not be affected during bending, twisting, and stretching. If the conductive layer is weakly bonded to the surface of the fabric or is not properly adhered to the surface, there is chances of flaking off from the conductive layer, affecting its electrical conductivity. Among different methods to coat the metal layer on fabric, there had been many attempts to use a method called physical vapor deposition (PVD) to evaporate metals and make thin conductive films on different types of fabrics [72]. Silver and copper layers have been deposited on polyethylene terephthalate (PET) yarns and polyurethane (PU)-coated nylon fabrics using sputtering techniques [73,74]. Silver is most suitable for coating due to its antibacterial property and high conductivity, but due to the fact that the weak adhesion of silver in frequently used fabric washing can be an issue; it can reduce the durability of the electrode and lower the electrical conductivity. Therefore, the surface modification of the textile substrate can better adhere the conductive materials onto it. Thiol group modified polyester fabric can provide high washability to the fabricated conductive textile electrode. It provides sufficient adhesion to the silver coated on the fabric by electroless plating. As silver has a high electrical conductivity, upon electroplating, an electrical resistance of 7.14 mΩ/square was obtained. The stability of the electrode upon stretching, washing, and oxidation was also checked. The resistance increased to 14.74 mΩ/square with 20% elongation, the conductivity was less affected by bending, and little of the silver layer fell off after 3000 bending cycles, as graphically represented in Figure 7. ECG signals were acquired by using thiol-grafted polyester coated with silver, stable ECG signals were acquired, and waveforms were distinguishable even after 200 washing cycles [75]. Forming a network on the fabric by crosslinking with a polymer also provides strong adhesion of the conductive material on top of the textile material other than surface modification. Coating the fabric with a mixture of silver nanowire and polyvinyl alcohol (PVA) and crosslinking the conductive layer with glutaraldehyde (GA) provide high reactivity between the aldehyde and hydroxyl group in cellulose and PVA. Using polymer for making the mixture and using a crosslinking agent fixes the silver nanowire on the fabric providing high washability. With three rounds of drop coating on the fabric, the electrical resistance increased, which was initially 0.3 ohm/square increased only up to 7.63 Ω/square for 100 h of washing. The ECG signals were distinguishable and stable for the electrode washed up to 60 to 80 h, with electrical resistance increasing up to 4.71 Ω/square [76].

### 4.2. Conductive Polymer Coating

**Figure 8:**
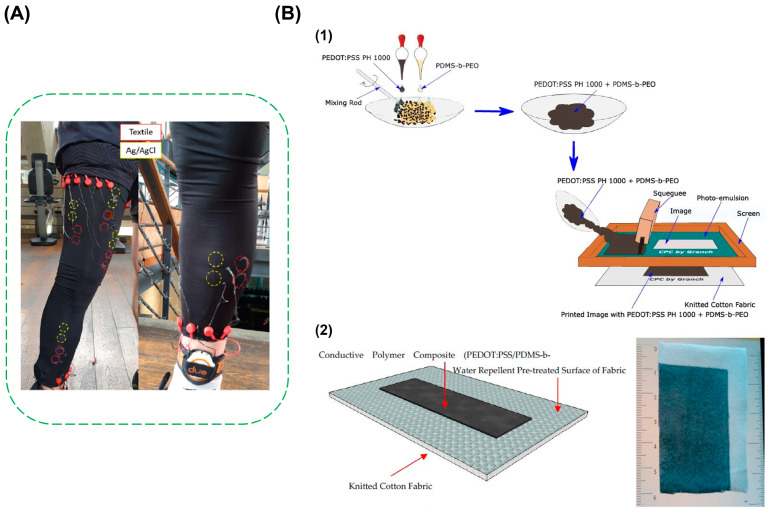
Different types of conductive polymers coated electrode. (**A**) Conductive electrodes attached to the leg sleeve worn by the subject. Marked in red is textile electrode and marked in yellow is Ag/AgCl electrodes. Adapted with permission from [80]. Copyright 2021, Spanu A et al. Published by IEEE. (**B**) (**1**) (the preparation of conductive polymer composite with PEDOT: PSS and PDMS-b-PEO and screen printing on knitted cotton fabric and (**2**) schematic representation of conductive composite layer on the fabric surface and the photograph of the actual conductive textile electrode. Adapted with permission from [81]. Copyright 2020, Tseghai G et al. Published by MDPI.

There is a certain class of organic polymers, which are electrically conductive owning to their structure. Poly(3,4-ethylenedioxythiophene)-poly(styrenesulfonate) (PEDOT: PSS), Polypyrrole (Ppy), and Polyaniline (PANI) belong to this category of intrinsically conductive polymers. These polymers have conjugated bonds with delocalized π-electrons giving them inherent property of electrical conductivity. The main limitation of the conductive polymers is their poor mechanical stability, stretchability, and flexibility [77]. This can be overcome by making composite of the conductive polymer with other elastic polymers and by using textiles as a substrate, which can result in high flexible conductive polymer composites [78,79]. PEDOT: PSS can be directly screen printed onto ready-made garments and used to obtain biopotential signals, such as EMG. To monitor the EMG signals from the leg, PEDOT: PSS can be screen printed onto stretched leg sleeves made of 100% polyester, given by Figure 8A. Pre-stretching the fabric before screen printing mimics the stretching of the garment when the subject wears it during the signal monitoring. This can help in retaining the electrical conductivity of the coating on the fabric while stretching. The validation of textile electrodes was done by placing both the textile electrodes and the Ag/AgCl electrodes on the same muscle. The electrical conductivity of the electrode was 73 ± 11 mS/cm. Coating the textile electrode with multiple layers of the conductive material increased the robustness of the electrode to mechanical stress [80]. To improve the mechanical robustness of the coating of conductive polymers on textile and to reduce its brittleness, a polymer composite can be made with a biocompatible elastic polymer along with the conductive polymers. Fabricating stretchable and flexible textile-based electrodes using polymer composite can improve the mechanical properties of the electrode, such as tensile stress. The fabrication steps for the preparation of PEDOT: PSS and PDMS-b-PEO composite and screen printing to fabricate a textile electrode is provided in Figure 8B [81].

### 4.3. Carbon and Its Derivatives

**Figure 9:**
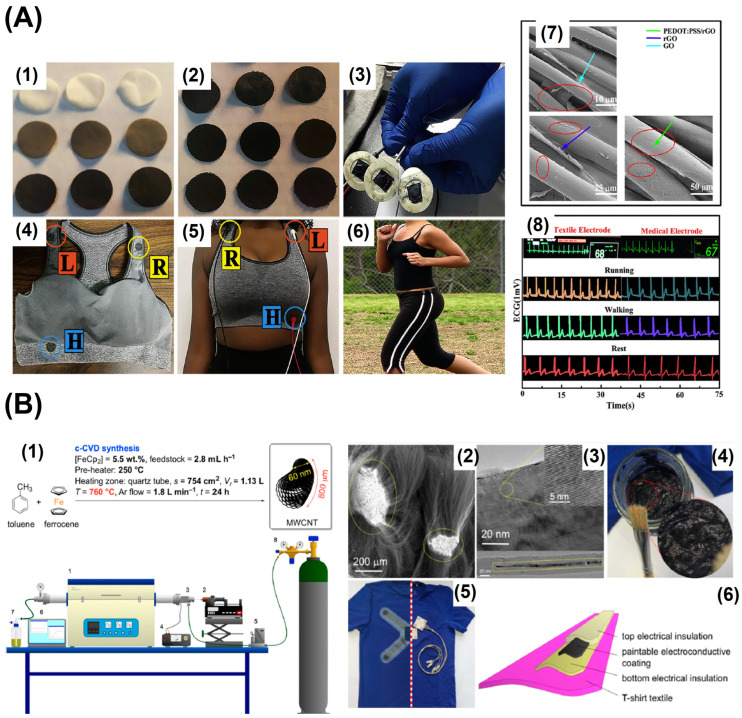
Different types of carbon and its derivatives as coated electrode. (**A**) Illustration of various stages of graphene-based breathable and washable textile electrode with pad–dry–cure method; (**1**) samples of cotton, GO coated and rGO coated textile fabric electrodes (**2**) P-rGO-1, P-rG)-2(DMSO) and P-rGO-3(EG) treated fabrics (**3**) conductive textile electrodes placed on metal probes connected to ECG device (**4**) placing and positioning of conductive textile electrodes in sports bra on left strap, right strap and bottom rib electrode (**5**) female volunteer student sports bra for ECG performance analysis in rest state (**6**) ECG performance analysis while running (**7**) SEM images of GO, rGO and PEDOT: PSS coated samples with different magnification (**8**) comparison of ECG acquired from fabricated graphene electrode and commercial Ag/AgCl electrode in different working conditions. Adapted with permission from [90]. Copyright 2020, Shathi et al. Published by Elsevier Ltd. (**B**) (**1**) Illustration of L-MWCNT synthesis by CVD (**2**) SEM images (**3**) TEM images of L-MWCNT (**4**) electroconductive paint made of L-MWCNT, 5 wt.% SDS and acrylic base (**5**) T-shirt with conductive layer painted (**6**) and geometry of the conductive layer on T shirt. Adapted with permission from [92]. Copyright 2022, Boncel et al. Published by American Chemical Society.

Due to their superior electrical and mechanical qualities, materials based on carbon have been extensively researched for use in sensing applications. They are desirable in the realm of biosensors and wearables due to their biocompatibility, flexibility, yield in higher quantities, and thermal and chemical stability [82,83]. Graphite, graphene, reduced graphene oxide, and carbon nanotubes are the mainly used carbon-based materials. Making textiles from these materials and using them go hand-in-hand. Because carbon materials can be produced in large quantities and are less expensive and because textile materials are readily available, therefore, there is a significant chance that commercialising the carbon-based textile electrode for electrophysiological signal monitoring will be possible if appropriate technologies are discovered that can generate a uniform stable coating of carbon on textile. Because of its biocompatibility and electrical conductivity, silver is one of the metals that is frequently used to create skin contact electrodes; however, due to its cost and tendency to oxidize, its potential use in greater quantities may be constrained. To create biocompatible skin contact electrodes, carbon materials, especially 2D materials such as graphene that are excellent conductors, can be a good choice [84]. Graphene can be used in a variety of ways to make textile materials conductible, including electrospinning, drop casting, and screen printing [85,86,87]. The simplest way to obtain a graphene-coated conductive textile is via dip coating or drop casting, which requires no special equipment. The graphene oxide dispersion, which was made using Hummers’ process, is applied to the nylon fibre. Hydrazine or hydrogen iodide can be used to reduce graphene oxide because it is less conductive. Nylon’s smooth surface, in contrast to materials such as cotton and Kevlar, makes it simpler for graphene to adhere to the fibre and create a good connection. After three iterations of dipping and drying, a sheet resistance of 13.96 kΩ/sq was attained. The manufactured electrode was incorporated into the calf band and utilised for monitoring EMG. In terms of signal acquisition, it performed on par with wet electrodes and had a higher signal-to-noise ratio [88]. Because of the toxicity of the reducing agent, utilising hydrazine to reduce the graphene oxide to reduced graphene oxide (rGO) is not an environmentally friendly process [89]. Ascorbic acid, for example, is a green reducing agent that can be used to convert GO chemically and thermally to rGO. The resulting rGO can then be applied to readily accessible, inexpensive textiles, such as cotton. Coating conductive polymers like PEDOT: PSS on top of the fibres with rGO coating will boost the conductivity of the electrode. These highly conductive, breathable electrodes can be fastened to active wearers, such as sports bras, making it very simple for women to monitor their ECGs while engaging in physical activity, represented in Figure 9A. The manufactured electrode is washable and did not degrade in the presence of sweat, which is inevitable during exercise [90]. When exercising, it is convenient to utilise a wireless ECG measurement gadget that can be attached on fabric. This enables the patient to move without being constrained by cables while wearing it. Different flexible materials can be used to make the interface electrodes for this wireless gadget. On a layer of thermoplastic urethane, MWCNT and PDMS composite were deposited to create one such flexible electrode. Initially, a layer of screen-printed silver was also applied, which resulted in an increase in the electrical conductivity from 77 S/m to 77,300 S/m. The electrodes were applied to the t-shirt’s chest region and heat pressed for appropriate adherence. Additionally, the impact of motion artefacts on the recorded ECG signals was examined. When compared to wet electrodes, the flexible dry electrode clearly caught the signal’s P wave. For a real-time signal analysis, the signals were sent over Bluetooth to an Android smartphone. Despite the waveforms being able to be distinguished, the quality of the signals obtained during exercise was less than that of the signals obtained at rest [91]. Another kind of wirelessly mountable electrode for long-term signal monitoring was created by adding a conductive layer directly to the fabric. Initially, an insulating layer was put on top of the fabric. To lower sheet resistance, the conductive CNT produced by CVD was coated multiple times on top of the insulating layer. The fabrication steps are detailed in Figure 9B. One of the most crucial tests that reusable textile electrodes had to satisfy was the testing of the coating’s performance under stress and washing. A gadget for continuous monitoring was created, and it operated continuously for over 24 h. The electrodes in this system feature insulated transmission without wires. During exercise, ECG signals were sent through snap fasteners to the recording device, but because the cloth is created conductive by individually painting a conductive layer on top of it, this process can restrict the possibilities of mass manufacturing [92]. The textile had to be customised for each patient because the precise position for positioning electrodes in the chest region can vary, which can affect the quality of the signals. This is one of the main drawbacks of the latter two methods for creating conductive textiles for wireless biopotential monitoring.

### 4.4. Direct Laser Writing

Laser-induced graphene (LIG) is a process that uses a laser to create a conductive pattern on a polymer substrate, which can be used as a sensing electrode. In recent years, there has been a growing interest in laser-induced graphene (LIG) obtained from polymers since its discovery in 2014 [93]. The LIG has gained attention as a promising material for biopotential electrodes due to its low-cost, flexibility, and biocompatibility. Lin’s research group has effectively detected various electrophysiological activities, such as electroencephalograms (EEGs), electrocardiograms (ECGs), and electromyograms (EMGs), by attaching mechanical sensors based on laser-induced graphene (LIG) to different parts of the human body [94]. Another study reported the development of a wearable LIG poly (3,4-ethylenedioxythiophene): poly (styrene sulfonate) (PEDOT: PSS)-based ECG electrode. The production of a reusable electrocardiography (ECG) dry electrode was achieved with direct writing of laser-induced graphene (LIG) onto a Kevlar textile [95]. The recent advancements in the development of LIG-based biopotential electrodes have shown promising results and provide a new avenue for the development of wearable and implantable biosensors.

### 4.5. Knitting, Weaving, and Embroidering with Conductive Threads, Yarns

**Figure 10:**
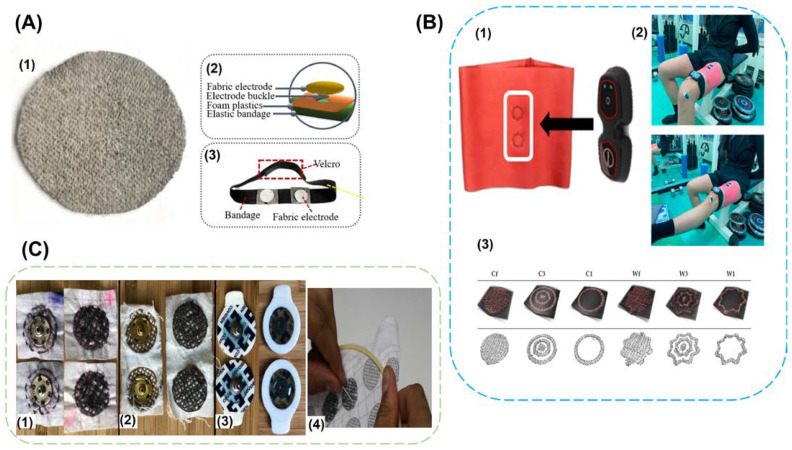
Different types of knitted, woven and embroidered textile electrodes (**A**) (**1**) image of silver-plated nylon filament woven fabric (**2**) assembly structure of fabricated electrode (**3**) electrode attached to a Velcro band. Adapted with permission from [97]. Copyright 2022, Zhang M et al. Published by MDPI. (**B**) (**1**) image representing the embroidered electrode and the wearable device which when attached to top of the embroidered pattern can be used to measure the EMG signals (**2**) images of actual EMG signal testing using the embroidered electrode as leg sleeve (**3**) various 3D images of circular and wavy embroidered pattern with the conductive yarn. Adapted with permission from [67]. Copyright 2022, Kim H et al. Published by MDPI. (**C**) Different hand made embroidered patterns (**1**) hand sewn embroidered electrode (**2**) machine sewn electrodes (**3**) gel electrodes for comparison (**4**) production of conductive textile by hand embroidery. Adapted with permission from [66]. Copyright 2020, Pitou S et al. Published by MDPI.

In addition to techniques that involve adding a separate layer of conductive coatings to create e-textiles, there are techniques through which textiles themselves can be made inherent conductive. The textile electrode’s metallic or carbon coatings may delaminate and lose conductivity as conductive pathways lose connectivity from repeated use and washing [65]. Since conductivity is included into the cloth during the weaving, knitting, and embroidery processes, there is no requirement for adhesives to hold the electrodes in place. Threads can be made conductive by adding conductive elements, including silver, copper, nickel, and carbon [96]. The electrode material, electrode size, thread spacing, and the density are the key variables that affect the quality of the collected biopotential signals [66]. Using a high density knit to make textile electrodes increases the surface contact area and perspiration accumulation, which can diminish the skin contact impedance. Due to its biocompatibility and antibacterial qualities, silver-coated yarns and threads are one of the materials that are most frequently utilised to create textiles. The ECG data were monitored using weaving patterns made from silver plated nylon filaments. The wet electrodes produced comparable results to those of the dry electrodes when the ECG signals were analysed both at rest and during dynamic motions. Figure 10A represents silver-coated nylon fabric and Velcro straps to which it is attached for placement on the human body [97]. Monitoring the EMG of the rectus femoris muscle in the leg was done utilising yet another conductive textile production technique that used technical embroidery equipment and silver yarn embroidery. An elastic sports garment was constructed with the electrode already built in, represented by Figure 10B. The signal quality is impacted by the stretching of the embroidered pattern and how it affects electrical conductivity. Hence, for this investigation, numerous embroidered patterns in a range of sizes were created. The electrode with a wavy design was stable during the stretching of the elastic cloth during physical motion and provided stronger EMG signals than the conventional circular pattern. Additionally, it was observed that the skin contact impedance decreased when increasing the density of the conductive yarn [67]. Since using embroidered conductive textiles for biopotential monitoring is a practical method, hand-stitched embroidery patterns were developed to cut the cost of production by doing away with the need for expensive embroidery machines and to be used as a local production technique in developing countries. The sensitivity of the hand-sewn electrodes was comparable to that of the machine-sewn electrodes. On polyamide cloth, conductive stainless-steel thread was used to embroider designs. To evaluate the quality of the textile electrode, electrical tests and measurements of muscle activity were performed. The hand-sewn electrode’s signal quality matched that of the conventional wet electrodes in a satisfactory manner. However, to ascertain the electrode’s reusability, it is also necessary to examine the fabric’s ability to endure stretching and washing. Various types of hand woven conductive textiles are represented in Figure 10C [66]. 

**Figure 11:**
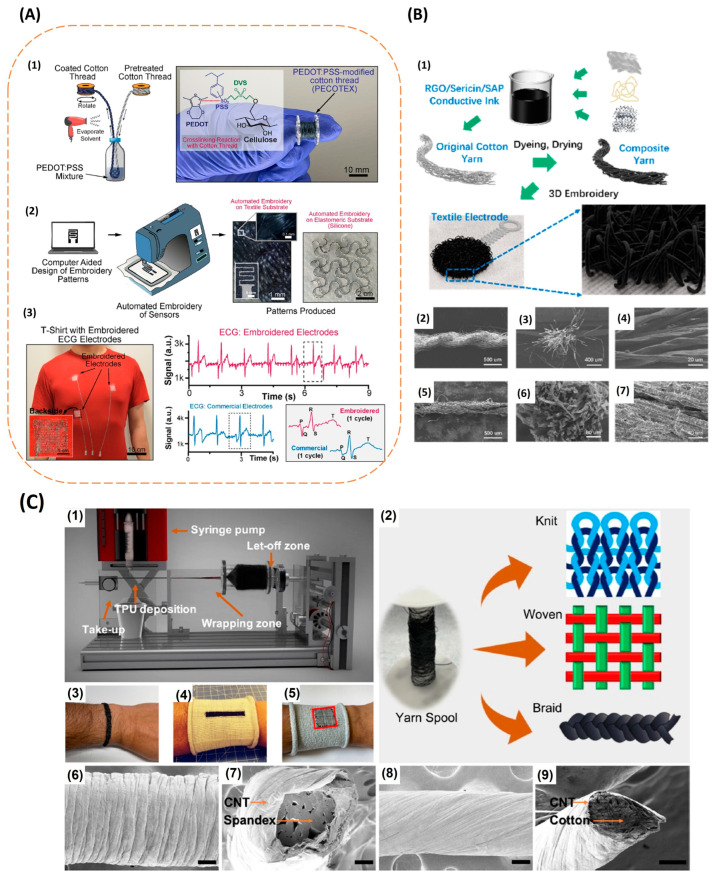
(**A**) Fabrication steps of computerized embroidered textile electrode (**1**) synthesis of PEDOT: PSS modified cotton thread and photograph of the produced conductive thread on bobbin (**2**) process of computerized embroidery on cotton fabric, patterns of interdigitated electrode produced on textile substrate and serpentine pattern produced on silicone substrate (**3**) T shirt with the embroidered electrodes, comparison of the ECG acquisition with embroidered electrode and commercial electrode. Adaptedwith permission from [65]. Copyright 2022, Alshabouna et al. Published by Elsevier Ltd. (**B**) (**1**) Illustration of fabrication steps for rGO coated conductive threads (**2**–**4**) SEM morphology of untreated cotton yarns (**5**–**7**) SEM morphology of conductive composite yarns. Adaptedwith permission from [98]. Copyright 2022, Elsevier Ltd. (**C**) (**1**) fabrication of CNT wrapped textile yarn (**2**) conductive yarn made into knitted, woven and braided structure (**3**–**5**) images of braided, knitted and woven electrodes sewn onto wrist band (**6**–**9**) surface and cross section morphology of CNT spandex, TPU and CNT coated yarn using SEM. Adapted with permission from [99]. Copyright 2022, Hossain et al. Published by Springer Nature.

Mass production of the wearable conductive textiles is essential since it less time consuming. Advanced technology like computerised embroidery can make this possible. However, this option is constrained by the unavailability of conductive thread that is compatible with the current conventional industrial textile mass manufacturing procedures. A new option for mass producing wearable electronic textiles has emerged with the use of a low-cost organic PEDOT: PSS-coated conductive thread with the mechanical robustness to be compatible with the current computerized embroidery technology. This technique was used to create a t-shirt that can track the ECG. The adherence of PEDOT: PSS to the OH-rich cotton fibre as well as the conductivity and mechanical strength of the conductive thread have all been improved by the combination of PEDOT: PSS, Ethylene Glycol (EG), and the crosslinker Divinyl Sulfone (DVS). The electrode’s capacity to be machine washed was examined. The electrical resistance was still stable after 15 washing cycles, and the ECG readings were on par with those generated by wet electrodes. During prolonged ECG monitoring, standard ECG waveforms were recognisable and stable. Figure 11A represents the fabrication steps involved in making the computerized embroidered textile electrode [65]. Because carbon materials have a high electrical conductivity, its many derivatives, such as reduced graphene oxide (RGO) and carbon nanotubes (CNT), have been used to create conductive yarns. To achieve greater skin conformity of the conductive yarn with the skin and thus lower skin contact impedance, 3D patterns were embroidered onto the cloth, given by Figure 11B. Flexible conductive yarns can be made conductive by dyeing and drying them in conductive ink composed of rGO, silk sericin, and a moisture-retentive substance. This electrode, which was woven into daily worn clothing, captured EMG and ECG signals of a high quality under both static and dynamic situations [98]. It was also possible to record high-fidelity ECG signals by braiding, knitting, and weaving cotton and spandex fibres wrapped with CNT. The manufactured conductive electrode passed the washability test and had a higher SNR than the wet electrodes. Knitted electrodes showed the highest stretchability and conformity of the three patterns, making them ideal for use in athletic wear and compression clothing. Figure 11C represents CNT-wrapped conductive yarns and its woven, braided, and knitted patterns [99].

## 5. Fabrication Methods of Conductive Textiles

### 5.1. Electrospinning

**Figure 12:**
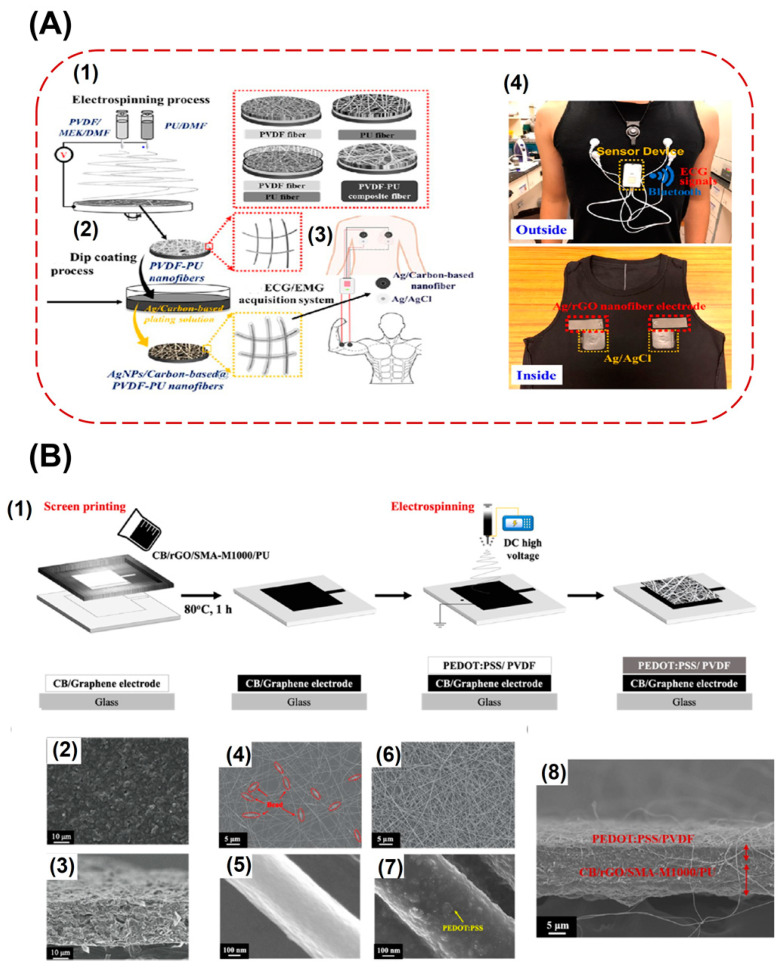
(**A**) (**1**) side by side dual nozzle electrospinning process of polymer nanofibers composed of polyurethane and polyvinylidene difluoride (**2**) steps illustrating the dip coating process of the PVDF-PU nanofibres into Ag/carbon based plating solution (**3**) ECG acquisition with the fabricat-ed electrode (**4**) Photograph of ECG smart clothing components along with the conductive nano-fibre membrane electrode using which ECG signals can be acquired and displayed on smart phone using Bluetooth based data transmission. Adapted with permission from [102]. Copyright 2022, American Chemical Society. (**B**) (**1**) schematic representation of nanofibre carbon electrode syn-thesis. SEM morphology (**2**) top surface of carbon electrode (**3**) cross sectional view of carbon elec-trode (**4**–**7**) fibre image and difference in morphology after adding PEDOT: PSS (**8**) cross section of nanofibre carbon electrode. Adapted with permission from [103]. Copyright 2021, American Chemical Society.

Electrospinning is a technique that is used to produce nanofibers. In this method, a strong electric field is used to get nanosized fibres [100]. Several types of electrodes can be prepared using this method. Polymer-based conductive electrodes can be prepared with the electrospinning method, and then, these electrodes can be attached to the ready-made daily worn garments and used to monitor the electrical signals from the body [16,101]. Elastic polymers, such as polyurethane and PVDF can be used to make nanofibers with electrospinning. With electrospinning, ultrathin nanofibers with diameter ranging from few micrometres to several hundred nanometres can be prepared using high voltage power supply; this method is simple and low-cost. These nanofibers have good membrane strength and high specific area, large porosity, and good mechanical strength. Dipping these electrospun nanofibers into a silver- and carbon-based conductive solution gives highly conductive nanofiber-based electrodes. Stable signals were obtained during high load activities during EMG. The fabrication steps in preparing electrospun conductive textile electrodes is represented in Figure 12A [102]. Instead of electrospinning elastic polymers alone, conductive polymers can also be used along with elastic polymers to get nanofiber electrodes. When electrospinning PEDOT: PSS along with PVDF, these nanofibers were deposited on carbon film during electrospinning. The presence of this electro spun conductive nanofiber layer on top of the conductive carbon layer increased the surface contact area of the electrode on the skin and enhanced the electrical conductivity. The fabricated flexible and stretchable nanofiber carbon electrode has low resistance, high tensile strength, good biopotential signal strength, and durability. They can be integrated on smart garments for the measurements. Steps illustrating the fabrication of nanofiber carbon electrode and the SEM images of the carbon electrode are represented in Figure 12B [103]. Electrospinning silk fibroin nanofibers on top of a non-woven PTFE fabric coated with silver nanowires can also impart adhesiveness to the electrode since silk fibroin is an excellent adhesive material. This electrode has a porous structure that gives breathability, electrical conductivity, wet skin adhesion, and surface hydrophobicity and hence can be used to obtain EMG and ECG signals [104].

### 5.2. Screen/Inkjet Printing

**Figure 13:**
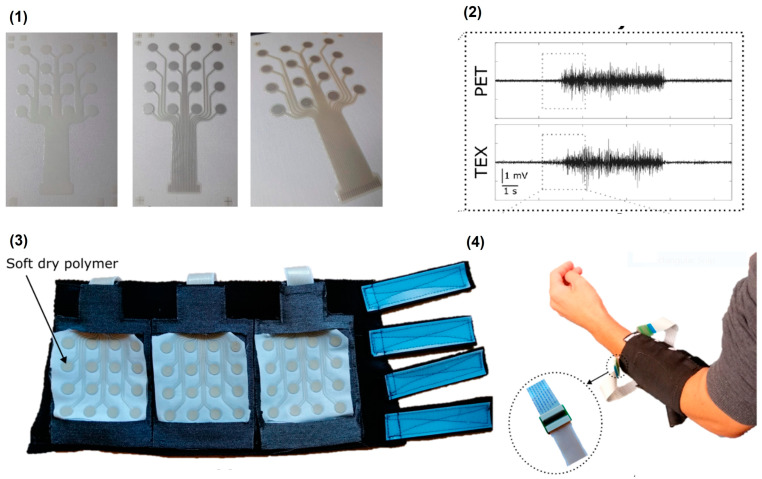
(**1**) Primer layer, conductive layer, and encapsulation layer screen printed on textile electrode (**2**) comparison of the EMG signals using both fabricated textile electrodes (**3**) fabricated textile electrode placed on the garment (**4**) integration of conductive textile electrode patches on hand sleeve. Adapted with permission from [68]. Copyright 2023, Murciego L et al. Published by MDPI.

Printing techniques are simple, fast, and easy techniques to print conductive coatings on fabric in large scale [105]. It can be used in industries since it can be used for large scale manufacturing. Different kinds of conductive inks can be used for printing on the fabric. Inks made of metal nanoparticles, conductive polymers, and carbon materials can be used for printing. The inks used for printing should be viscous and should strongly bind to the surface of the fabric. It should not flake off or lose the conductivity when the fabrics undergo stretching, washing, and reusing [106]. Screen printed electrodes can be used for gesture controlling. First, conductive layers are printed with a silver polymer ink for conductive tracks. The final layer is a stencil printed conductive carbon rubber paste. Silicon rubber is also provided around the conductive layer to provide improved adhesion to the fabric. The electrodes are placed on the specific muscles. Three muscular groups were targeted, such as the brachioradialis, extensor group on the outer forearms, and the flexor group on the inner forearm [107]. The surface of the fabrics can also be modified using thermal transfer technology by coating it with a polymer layer to make the surface of the fabric rough and for better binding of the conductive materials to the fabric surface. Screen printing patterns were made on an untreated textile substrate and on top of modified textile. Viscous graphene ink is screen printed on the fabric, has comparable performance with wet electrodes during ECG acquisition, and has good stability after multiple washes, and its resistance increases with an increase in the number of washing cycles. This can be due to water absorption and small graphene nanoflake loss [108]. The use of three-layer printing with screen printing has been utilised to achieve a continuous conductive route and prevent any printing defect-related reduction in the performance of the textile electrode. It starts with printing a priming layer to smooth the surface, followed by printing the conductive layer and an encapsulating layer to protect the conductive tracks from electrical currents, as represented in Figure 13. For optimal skin-to-electrode contact, a dry skin–electrode interface is covered with a soft, flexible, conductive medical grade polymer. The performance of the electrode was compared to that of a standard electrode printed on a PET film covered in hydrogel in order to benchmark this three-layer printing process. On a PET film covered in hydrogel in order to benchmark this three-layer printing process. With the two electrodes, comparable EMG signal quality, signal to noise ratio, frequency spectra, and spatial distribution were found. The absence of motion artefacts in the recorded signals indicates that the soft polymer used absorbed minor skin deformations [68].

Another printing method with high-quality and an effective output is inkjet printing [109]. Directly creating conductive layers on the fabric surface is possible with the use of computer technology. A conductive layer can be formed on the surface of a cloth at ambient temperatures by combining inkjet printing technology with a new liquid phase chemical reduction method. With this technique, textiles are not exposed to higher temperatures for sintering. A fabric surface that has been moistened by ascorbic acid is treated with aqueous silver nitrate using a piezoelectric droplet ejection device. Conductive layers are immediately formed at the surface of a redox reaction. According to SEM morphology, the fabric’s pores have been filled with conductive elements, increasing the conductivity. Compared to bulk silver, the printed conductive layer demonstrated a greater conductivity. Different stretch orientations on the knitted textiles were used to assess the resistance variance [110].

### 5.3. Drop/Dye/Dip Coating

Dip coating, drop coating, and dye coating are very simple and low-cost techniques that can be used for the fabrication of conductive textiles for biosensing applications. Conductive polymers, conductive solutions, etc. can be coated on the textile surface by this method [111]. Conductive polymers like PEDOT: PSS mixed with DMSO to enhance the conductivity can be coated on top of cotton fabric with a simple drop coating method. ECG signals obtained from the conductive cotton electrodes were found to be stable [112]. These easy one-step methods can also be used to coat carbon-based materials on textile. Dyeing the fabric with graphene oxide and reducing it gives a conductive reduced graphene oxide-coated textile. Dip coating the fabric into conductive polymer PEDOT: PSS with a layer-by-layer technique enhances the conductivity of the already conductive fabric. There was decrease in sheet resistance from 2.5 MΩ to 120 Ω upon dip coating PEDOT: PSS to rGO-coated fabrics. The textile was stable under 20–30 washing cycles, and high-quality ECG signals were obtained in both wet and dry conditions [113].

A summary on the advantages and disadvantages of textile based dry electrodes from recently published articles are given in Table 1 below.

## 6. Essential Characteristics for a Wearable Textile Electrode

**Figure 14:**
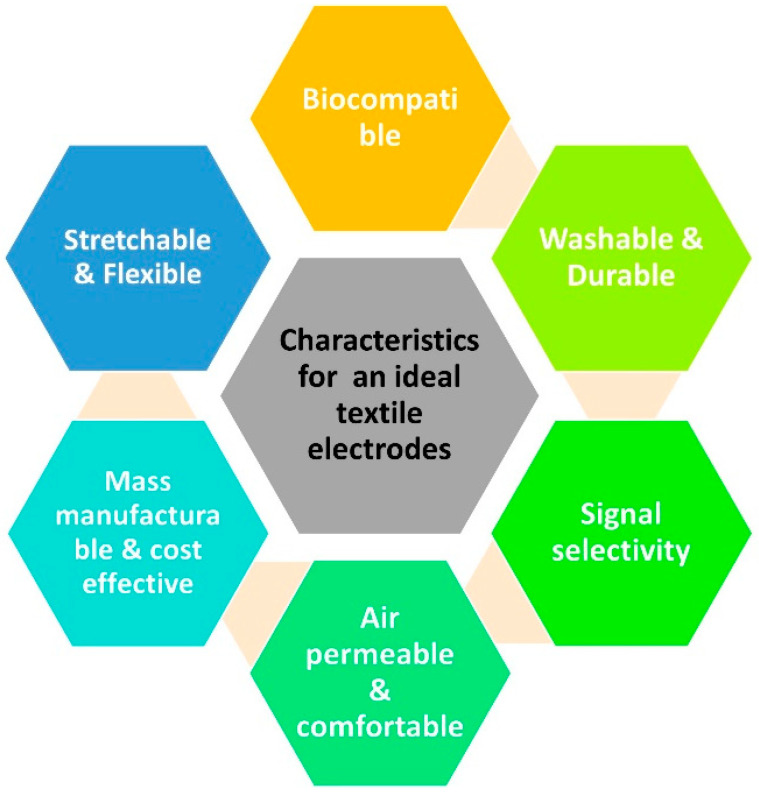
Characteristics required for an ideal textile electrode.

To make conductive textile electrodes for biopotential signal monitoring more effective than currently available gel electrodes in terms of signal quality and comfort as well as to enable their commercialization, research into their fabrication and modification is accelerating. Textile electrodes are increasingly being used for electrocardiogram (ECG) and electromyogram (EMG) monitoring due to their many advantages, such as improved comfort, reduced motion artifacts, and better adherence to the skin. The benefits that textile electrodes provide for comfortable long-term ECG signal monitoring at home or for tracking muscle activity during muscle training for athletes are unquestionably improvements in wearable technologies for the growth and advancement in the healthcare sectors [123,124]. To be employed as biopotential signal monitoring electrodes, textile electrodes must meet a number of qualitative and diagnostic requirements in order to be commercially viable and trusted by consumers. The choice of conductive materials should be significant since textiles act as insulation. Low surface resistance, homogeneous conductivity, and biocompatibility with human skin are all requirements for the conductive material [125,126]. The skin electrode contact may be unstable since there is no gel on the interface [46]. To reduce the skin’s contact impedance, a variety of techniques can be used, including contact pressure and ionic gels [19,126,127]. An impedance analyzer can be used to measure the electrodes’ average impedance. A silver yarn textile electrode’s average impedance was tested in a study by Katherine et al., and it was discovered to be less than 2 k, satisfying the ANSI/AAMI standard requirement with a frequency range of 20 Hz to 10 kHz [128]. Obtaining distinct and clear P wave, QRS complex, and T wave ECG waveforms is one of the diagnostic qualities. The obtained signals should have the least amount of noise possible. Power spectral density can be used to determine the frequency components that the electrode has detected [32,129]. The performance of the textile electrode in picking up low frequency signals must be tested because biopotential signals are in the low frequency range. Tensile strength, flexibility, and stretchability are further significant requirements that the textile electrode should meet [52,130,131]. A representation of the required characteristics for an ideal textile electrode is given is Figure 14.

A good textile electrode should possess certain characteristics to ensure the optimal performance for ECG and EMG monitoring [123,130].

- Biocompatibility: The electrode should be made of materials that are safe for the skin and do not cause any irritation or allergic reactions;
- Flexibility: Textile electrodes should be flexible and able to conform to the contours of the body for comfortable and accurate placement;
- High conductivity: The electrode should have low impedance to ensure good signal quality and reduce noise;
- Stability: The electrode should be stable over time and not deteriorate or lose its conductivity during use;
- Durability: The electrode should be able to withstand repeated use and washing without damage;
- Cost-effectiveness: The electrode should be affordable and cost-effective for widespread use in healthcare and research applications;
- Ease of use: The electrode should be easy to apply and remove and not require specialized skills or training;
- Compatibility: The electrode should be compatible with standard ECG and EMG equipment for seamless integration into existing systems;
- Signal selectivity: The electrode should have high signal selectivity for specific biopotential signals, such as ECG or EMG, to avoid crosstalk and interference from other sources;
- Washability: The electrode should be washable to allow for repeated use and maintain good signal quality after washing.

### 6.1. Electrode Design

The electrical performance of the conductive textile will vary depending on the manufacturing design of the fabric, such as woven or knitted. Brehm et al. used an iterative MATLAB optimizer in their work to investigate the effects of the woven fabric pattern, yarn type, and surface area on electrical conductivity. It was found that the more space there was, the more noise there was as well as larger signal amplitude waveforms. Although resistance varies depending on the type of conductive yarn, there was little variation in capacitance with the type of yarn [132]. Most of the previous studies have shown that the larger the electrode is in diameter, the better the signal quality and the lower the skin contact impedance [133]. In addition, significant contributions of distortions to the recorded ECG or EMG signals in the lower frequency range are minimized when electrodes of larger diameters are used [18]. Though there is relationship between electrode size and signal quality, other factors like electrode material and its electrical conductivity also affects the signal quality [134].

### 6.2. Effect of Pressure on the Electrode

Application of pressure on the electrodes has a strong influence in the signal quality. The optimum contact pressure is needed to reduce motion artefacts since the absence of a gel layer can make the contact between the electrode and the skin unstable [19]. In many textile-based electrode designs, the conductive electrodes are embedded onto tight fitting elastic garments, such as sports wears, or attached to elastic bands that can be wrapped around the chest, arms, or legs [133,135] Using elastic, tight-fitting garments ensures sufficient pressure is applied onto the garment, and the comfort of the subjects wearing them should also be taken into consideration since garments that are too tight can cause discomfort to the subjects, especially during long-term monitoring. The optimum clothing pressure comfortable to the wearer with comparable performance with wet electrodes was investigated in several studies [136]. In a study by Takeshita et al., the ideal pressure to apply for the patient to avoid motion artefacts was discovered to be between 1000 Pa and 2000 Pa. For very minor displacement of the skin under the fabric, the ECG signals recorded under this pressure have no motion artefacts [137]. Due to the absence of a gel layer, the electrodes are prone to small displacement with the movement of the patient, which can affect the electrical properties at the interface and induce motion artefacts. The effect of pressure on reducing the motion artefact and lowering skin contact impedance was investigated in few studies, and it was found that applying an optimum pressure on the electrode has a positive outcome [19].

### 6.3. Effect of Noise and Motion Artifacts

The key benefit of employing textile electrodes built into T-shirts and other intelligent clothing is that they may be worn for longer periods of time to monitor the activity of the heart in circumstances like arrhythmias [34,35]. Commercially available equipment, such as the Holter ECG machine, can be used for conventional methods of long-term heart rate monitoring; however, because of its lengthy wires, it may restrict the patient’s movement. ECG signals can be measured without interfering with the patient’s everyday activities when employing smart clothing with electrodes and wireless modules integrated into it for heart rate monitoring. This provides the patient with a great deal of comfort, but the patient’s increased activity might also result in more noise and negative effects in quality of ECG signal. In a study by David et al., the performance of the Ag/AgCl electrode and electrodes constructed of silver yarn integrated into textiles was compared. Signals acquired using both approaches in the resting state were comparable while the ECG signals in both approaches during the dynamic period were of lesser quality. Both techniques had trouble distinguishing P waves. This study found that neither strategy outperformed the other in dynamic conditions [138]. In another study by Le K et al., how well gel electrode performed was compared to a silver-based textile electrode. Assessments that are both qualitative and diagnostic have been carried out. The amplitude of the ECG signals was seen higher for a gel electrode than the textile electrode, though it did not affect the heart rate determination. When the Pearson correlation coefficient was calculated, it was discovered that the two-electrode material performed similarly. When comparing the power spectral densities of textile and gel electrodes, it was discovered that both exhibited the same trend in the spectral density graph [129].

During ECG and EMG measurements, several types of artifacts or interference can be observed, which may affect the quality and accuracy of the recorded signals. Some of the most common types of artifacts encountered in ECG and EMG measurements include motion artifacts, electrode motion artifacts, power line interference, and baseline drift [139,140]. The motion artifacts can arise from patient motion, poor electrode contact, electrical interference from other equipment, and various physiological factors. The level of artifacts should be kept as low as possible with proper electrode placement, subject at a relaxed position with minimum motion state, and other equipment kept off while ECG monitoring [141]. Motion artifacts in ECG can be monitored through various methods, including visual inspection, signal processing techniques, and machine learning algorithms. Xie et al. proposed a multi-stage ECG denoising framework concentrating on the detection of motion artifact based on domain knowledge. The motion artifacts are located with a noise-adaptive thresholding [142]. Lee et al. presented an automatic motion and noise artifact detection in Holter ECGs using EMD and statistical features, such as Shannon entropy, mean, and variance of the first intrinsic mode function (IMF) [143]. Kim et al. proposed a two-stage cascade adaptive filtering for efficient motion artifact removal, where a noise reference signal is required to estimate the characteristics of motion artifact [144].

A power line interference occurs when the electrical activity from power lines interferes with the recorded signal, resulting in 50 or 60 Hz noise. A baseline drift can occur due to changes in electrode–skin contact, temperature, or humidity and can cause slow changes in the recorded signal over time [30].

Several methods have been proposed in different articles to remove these artifacts from the recorded signals. These include filtering techniques, such as bandpass filters, notch filters, and adaptive filters as well as signal averaging, wavelet transforms, and independent component analysis (ICA) [30,141,145]. Some studies have also explored the use of advanced machine learning techniques, such as deep neural networks, for artifact removal. For instance, a recent study proposed an automated deep learning approach for removing motion artifacts from ECG signals [146]. The study used a convolutional neural network (CNN) to identify and remove motion artifacts from the ECG signal. Another study has shown that sinusoidal signal modelling is an effective approach to removing power line interference (PLI) and baseline wonder (BW) from ECG and EMG signals [147]. Overall, various approaches have been explored to remove artifacts and interference from ECG and EMG signals, with the choice of method depending on the type and severity of the artifact and the specific application of the recorded signals.

### 6.4. Effect of Washing and Stretching

The reusability and durability of textile electrodes are two major issues that must be resolved among the other requirements that textile electrodes must meet in order to be used for long-term ECG monitoring. Sweat can be a problem with them since they are being used during exercises and also in long-term monitoring. The electrodes should be washable, and washing them should not cause the conductive coatings on the textile’s surface to peel off, which would reduce the electrodes’ electrical capabilities [148]. Although stretching is likely to occur when utilising textile electrodes during exercise, this should not have an impact on the electrode’s electrical conductivity or capacity for signal acquisition.

Zaman et al. evaluated the washability of various textile electrode types used for ECG monitoring, their degradation, and loss of electrical properties with multiple washing cycles as they can suffer stretching, bending, and twisting during washing. The washability of copper-coated fabrics knitted fabrics with silver plating, and embroidered electrodes were tested. After 50 washing cycles, it was found that the performance of the silver-coated electrodes remained consistent while that of the copper-coated electrodes increased because less copper was adhering to the fabric’s surface. This study included further studies to investigate the impact of water and detergent on conductive textiles. Silver-plated knitted materials did not exhibit any performance deterioration [20].

After washing, textile electrodes with conductive coatings on top may experience higher surface resistance as a result of the coating flaking off due to poor adherence [69]. However, by improving the attachment of conductive materials to the surface of textile electrodes, the stability of the electrode during washing can be increased [75]. The direction of the stretch in relation to the fibre determines stretchability and its impact on electrical conductivity [149,150].

## 7. Challenges and Limitations

Dry textile electrodes have gained considerable attention in recent years due to their ease of use, comfort, and portability compared to conventional wet electrodes. However, they also have several challenges and limitations that need to be addressed for widespread adoption in ECG and EMG monitoring applications [46,123]. These challenges and limitations include:

- Poor signal quality: Dry textile electrodes have higher skin–electrode impedance than wet electrodes, resulting in poor signal quality and increased noise in the recorded signals;
- Limited electrode-skin contact area: Dry electrodes rely on mechanical pressure to maintain good contact with the skin, which can be challenging to achieve consistently over time. This limited electrode–skin contact area can result in poor signal quality and inconsistent recording of biopotential signals;
- Sensitivity to motion artifacts: Dry electrodes are more sensitive to motion artifacts than wet electrodes, as they rely solely on skin–electrode contact for signal acquisition. Movement or muscle contractions can cause the electrode to lose contact with the skin, leading to signal loss or artifacts;
- Lack of standardization: Currently, there is no standardized design or manufacturing process for dry textile electrodes, resulting in variability in electrode performance and compatibility with existing ECG/EMG equipment;
- Limited lifespan: for some dry electrodes have a limited lifespan due to the wear and tear of the conductive materials and the adhesive used to attach them to the skin. This can result in decreased signal quality over time and increased replacement costs;
- Crosstalk and interference: Dry electrodes may be susceptible to cross-talk and interference from other sources, such as electrical noise from nearby devices or from other electrodes on the same individual;
- Biodegradability: Some of the primary environmental concerns, such as biodegradability, are being addressed by growing trends towards the manufacturing of green textile electrodes. Cotton is one of the more popular substrates for making textile electrodes. They are biodegradable, soft, and flexible [99]. Polyester-based textile electrodes are not biodegradable and can pose a risk to the environment [151,152];
- Addressing these challenges and limitations is critical for the widespread adoption of dry textile electrodes in ECG and EMG monitoring applications. Ongoing research is focused on developing new materials, designs, and manufacturing processes to overcome these limitations and improve the performance of dry textile electrodes for biopotential monitoring.

## 8. Summary and Conclusions

In conclusion, wearable biopotential signal monitoring is an important tool in daily life for the early detection and monitoring of various health conditions. Textile electrodes have emerged as a promising technology for wearable biopotential signal monitoring. Recent advances in the development of textile electrodes have shown great potential for improving the accuracy and reliability of biopotential signal measurements and enhancing the usability and accessibility of wearable biopotential signal monitoring and reducing healthcare costs. However, there are still challenges that need to be addressed, and further research is needed to overcome these challenges and realize the full potential of textile electrodes for wearable biopotential signal monitoring. This comprehensive review paper provided a valuable resource for researchers, clinicians, and industry professionals working in this area, highlighting the basics of biopotentials to recent advances and challenges in the development of textile electrodes for biopotential signal monitoring.

## Figures and Tables

**Table 1:** Comparative analysis of the different varieties of textile electrodes.

References	Textile Electrode Type	Fabrication Method	Benefits	Drawbacks
[114] Yang et al. (2023)	Metal based electrodes	Dip coating fabric into Ga- liquid metal particles solution	Conductive patterns heals automatically when cut, permeability, conductivity, easy to fabricate, antibacterial property	Coating of textile as a whole, selective region coatings can be explored
[115] Lim et al. (2023)	Metal based electrodes	Ga-liquid metal particles spray coated on nylon/spandex fabric and was immersed in aqueous Au solution	Good electrical and mechanical stability under stretching, good adhesion between conductive layers	Using gold can increase the cost of fabrication
[116] Qian et al. (2022)	Metal based electrodes	Ag particles deposited on polyester fabric by electroless plating	High washing stability, electrical stability even after 500 washing cycles	Biodegradability can be a concern for the polyester fabric
[112] Maithani et al. (2022)	Conducting polymer	Coating of PEDOT:PSS on the fabric	Good electrical conductivity, easy to fabricate	Loose their mechanical property during wet conditions
[117] Jain et al. (2023)	Conducting polymer	Bio-conductive of modified cellulose fibers and PEDOT: PSS prepared for 3D printing	Printed patterns have good conductivity at low PEDOT: PSS concentration, high tensile strain	Washing stability and reusability has to be explored if to be used in wearables
[118] Ohiri et al. (2022)	Conducting polymer	Dip coating polyester fabrics into PEDOT: PSS solution	Conductive textiles incorporated into compressive garments can be machine laundered, resistance to high strain, rapid prototyping possible	Use proprietary materials for conductive surface (Dupont CCSM)
[119] Dong et al. (2022)	Carbon-based electrodes	Dip coating of electrospun woven fabric into CB/CNT mixture	Ultra stretchable, self healing non woven fabric, long term use stability under harsh environment	Reusability and skin contact impedance can be studied
[120] Ali et al. (2023)	Carbon-based electrodes	Graphene NP/PVDF mixture cast and cured on polyester	Reusable, biocompatible, non irritant to skin, flexible	High temperature involved in the synthesis procedure
[121] Guler et al. (2022)	Carbon-based electrodes	Three step print-dry-reduce spray painting process to coat graphene on textile	ECG acquisition from region behind the ear using this soft graphene coated textile, High SNR 29.87 dB	Prone to motion artefacts when the electrodes move, need to explore this by postprocessing of the recorded ECG
[122] Etana et al. (2023)	Embroidered conductive threads electrodes	Computerized embroidery machine used to make conductive fabric with polyester multifilament with conductive hybrid thread	Comparison of optimum pressure and the signal quality	EMG signals were affected by motion artefacts

## Data Availability

Not applicable.
